# Intensive care unit delirium is an independent predictor of longer hospital stay: a prospective analysis of 261 non-ventilated patients

**DOI:** 10.1186/cc3729

**Published:** 2005-06-01

**Authors:** Jason WW Thomason, Ayumi Shintani, Josh F Peterson, Brenda T Pun, James C Jackson, E Wesley Ely

**Affiliations:** 1 Attending Physician, Division of Allergy/Pulmonary/Critical Care Medicine, Vanderbilt University School of Medicine, Nashville, TN, USA; 2 Research Assistant Professor of Biostatistics and Medicine, Departments of Internal Medicine, Divisions of General Internal Medicine and Center for Health Services Research, Vanderbilt University School of Medicine, Nashville, TN, USA; 3 Assistant Professor of Medicine and Bioinformatics, Departments of Internal Medicine, Divisions of General Internal Medicine and Center for Health Services Research, Vanderbilt University School of Medicine, Nashville, TN, USA; 4 Clinical Assistant Professor of Nursing, Division of Allergy/Pulmonary/Critical Care Medicine, Vanderbilt University School of Medicine, Nashville, TN, USA and Center for Health Services Research, Vanderbilt University School of Medicine, Nashville, TN, USA; 5 Research Assistant Professor of Medicine and Psychiatry, Division of Allergy/Pulmonary/Critical Care Medicine, Vanderbilt University School of Medicine, Nashville, TN, USA and Center for Health Services Research, Vanderbilt University School of Medicine, Nashville, TN, USA; 6 Associate Professor of Medicine, Division of Allergy/Pulmonary/Critical Care Medicine and Center of Health Services Research, Associate Director of Research, VA Tennessee Valley Geriatric Research, Education and Clinical Center (CRECC), Vanderbilt University School of Medicine, Nashville, TN, USA

## Abstract

**Introduction:**

Delirium occurs in most ventilated patients and is independently associated with more deaths, longer stay, and higher cost. Guidelines recommend monitoring of delirium in all intensive care unit (ICU) patients, though few data exist in non-ventilated patients. The study objective was to determine the relationship between delirium and outcomes among non-ventilated ICU patients.

**Method:**

A prospective cohort investigation of 261 consecutively admitted medical ICU patients not requiring invasive mechanical ventilation during hospitalization at a tertiary-care, university-based hospital between February 2002 and January 2003. ICU nursing staff assessed delirium and level of consciousness at least twice per day using the Confusion Assessment Method for the ICU (CAM-ICU) and Richmond Agitation-Sedation Scale (RASS). Cox regression with time-varying covariates was used to determine the independent relationship between delirium and clinical outcomes.

**Results:**

Of 261 patients, 125 (48%) experienced at least one episode of delirium. Patients who experienced delirium were older (mean ± SD: 56 ± 18 versus 49 ± 17 years; p = 0.002) and more severely ill as measured by Acute Physiology and Chronic Health Evaluation II (APACHE II) scores (median 15, interquartile range (IQR) 10–21 versus 11, IQR 6–16; p < 0.001) compared to their non-delirious counterparts. Patients who experienced delirium had a 29% greater risk of remaining in the ICU on any given day (compared to patients who never developed delirium) even after adjusting for age, gender, race, Charlson co-morbidity score, APACHE II score, and coma (hazard ratio (HR) 1.29; 95% confidence interval (CI) 0.98–1.69, p = 0.07). Similarly, patients who experienced delirium had a 41% greater risk of remaining in the hospital after adjusting for the same covariates (HR 1.41; 95% CI 1.05–1.89, p = 0.023). Hospital mortality was higher among patients who developed delirium (24/125, 19%) versus patients who never developed delirium (8/135, 6%), p = 0.002; however, time to in-hospital death was not significant the adjusted (HR 1.27; 95% CI 0.55–2.98, p = 0.58).

**Conclusion:**

Delirium occurred in nearly half of the non-ventilated ICU patients in this cohort. Even after adjustment for relevant covariates, delirium was found to be an independent predictor of longer hospital stay.

## Introduction

Delirium is defined as an acute change or fluctuation in mental status plus inattention, and either disorganized thinking or an altered level of consciousness at the time of the evaluation [1,2]. Numerous studies have described the incidence, prevalence, and costly impact of delirium with regard to patients in nursing homes and hospital wards [3-7], but few prospective investigations have focused on cohorts treated specifically within the intensive care unit (ICU). Several studies have now confirmed that delirium occurs in 60% to 80% of mechanically ventilated patients [2,8-10], though two investigations found a lower prevalence in an ICU cohort with a lesser severity of illness [11,12]. Among ventilated patients, this condition is independently associated with untoward clinical outcomes [10,13], including higher mortality [10]. In fact, every day spent in delirium was associated with a 10% higher risk of death and worse long-term cognitive function [10].

Only 5% of 912 critical care professionals surveyed in 2001 and 2002 reported monitoring for ICU delirium [14], and yet the Society of Critical Care Medicine (SCCM) has recommended routine monitoring for delirium for all ICU patients [15]. Because many aspects of delirium in the ICU may be preventable and/or treatable (e.g., hypoxemia, electrolyte disturbances, sleep deprivation, overzealous use of sedative agents), routine daily delirium monitoring may be justified in non-ventilated ICU patients if adverse outcomes were demonstrated among delirious patients within this population.

Therefore, we undertook this investigation to determine the incidence of delirium among non-ventilated ICU patients and to determine the association between delirium and length of stay in the ICU, length of stay in the hospital, and in-hospital mortality.

## Materials and methods

### Patients

The institutional review board at Vanderbilt University Medical Center (Nashville, TN, USA) approved this observational cohort study [16] as Health Insurance Portability Accountability Act compliant, and informed consent was waived. Enrollment criteria included any patient aged 18 years or older who was admitted for more than 24 hours to the medical ICU of Vanderbilt University's 658-bed medical center, and who did not require invasive mechanical ventilation. During the 11-month study interval from 1 February 2002 to 7 January 2003, all of the 261 patients who met the inclusion criteria were enrolled in the study and followed until either death or hospital discharge. None of the patients in this cohort have been previously published in other peer-reviewed manuscripts.

### Data collection and study design

Nursing staff assessed sedation level via the Richmond Agitation-Sedation Scale (RASS; see Additional file 1) [17,18] and delirium status via the Confusion Assessment Method for the Intensive Care Unit (CAM-ICU; see Additional file 2) as described in previous literature [2,19] (downloadable materials and discussion also available at [20]). Of note, the CAM-ICU has been validated in both non-ventilated and ventilated patient assessments [2,19]. These data were recorded prospectively at least once per 12-hour shift as part of routine nursing care. The implementation of delirium monitoring in our institution took place through a year-long quality assurance program. During this time, the validity and inter-rater reliability of the RASS and CAM-ICU were very high [16] and consistent with our previous reports [2,18]. Specifically, the compliance was 90% in over 2,000 patient bedside observations and agreement with reference standard CAM-ICU raters was high (kappa = 0.80). Information collected prospectively at the time of enrollment included patient demographics, severity of illness using the Acute Physiology and Chronic Health Evaluation II (APACHE II) [21] score, and admission diagnoses. The Charlson Comorbidity Index, which represents the sum of a weighted index that takes into account the number and seriousness of pre-existing co-morbid conditions, was calculated using ICD-9 codes as per Deyo *et al*. [22]. The diagnostic categories for ICU admission were recorded by the patients' medical teams as the diagnostic category most representative of the reason for ICU admission. Because this was not an intervention study, no specific treatment(s) were given to patients who were identified as delirious. All therapies with regard to sedation and delirium were left to the discretion of the physician team caring for each patient.

Delirium in the ICU was the independent variable for this study and was classified as in previous reports [9,10]. Patients who scored positive for delirium by the CAM-ICU at any time while in the ICU were categorized as 'Ever Delirium'. All others were categorized as 'Never Delirium'. The three dependent variables included lengths of stay in the ICU and in the hospital, and in-hospital mortality.

### Statistical analysis

Fisher's exact tests, exact chi-square tests, and Wilcoxon rank sum tests were used as appropriate to determine whether or not baseline features differed between those with and without delirium. Cox proportional hazards regression analyses [23] were used to assess the effects of delirium on ICU length of stay, hospital length of stay, and time to in-hospital mortality. In order to conduct the most robust analysis of the relationship between delirium and the outcome variables, delirium was included as a time-dependent incidence variable, and coded as 0 for all days prior to the first delirious event and as 1 thereafter. Coma status was also included in each model as a time-dependent covariate and was coded similarly. Other baseline covariates included in each model were age, gender, race, APACHE II score, and Charlson co-morbidity index. Because of the limited number of events for the time to in-hospital mortality analysis, and in order to avoid consequences of over-fitting that might have resulted from including each covariate separately, principal component analysis was used to pool the effects of age, gender, race, APACHE II score, and Charlson for the mortality analysis only. Time-to-event curves were created according to the methods of Kaplan and Meier [24], and were compared using log-rank tests. All statistical analyses were conducted using SAS Release 8.0.2 (SAS Institute, Cary, NC, USA).

## Results

### Baseline characteristics

Of the 261 patients enrolled in the study, 125 (48%) experienced delirium. One patient was excluded from analysis because of persistent coma throughout the entire hospital stay, negating any attempts to define the presence or absence of delirium. Baseline characteristics of the patients are presented in Table 1, with the cohort divided into two groups: Ever Delirium (n = 125) and Never Delirium (n = 135). There were no significant differences between the Ever Delirium and Never Delirium groups for gender, race, Charlson co-morbidity scores, or admission diagnoses. The Ever Delirium patients were significantly older (mean 56 versus 49 years of age, p = 0.002), and had higher APACHE II scores (median 15 versus 11, p < 0.001). Primary medical diagnoses were similar between the groups, with pulmonary (e.g., chronic obstructive pulmonary disease exacerbation), gastrointestinal (e.g., variceal hemorrhage), and metabolic (e.g., drug overdose, diabetic ketoacidosis) syndromes being the most common reasons for admission to the ICU.

### Clinical outcomes and multivariable analysis results

#### Lengths of stay

**Figure 1:**
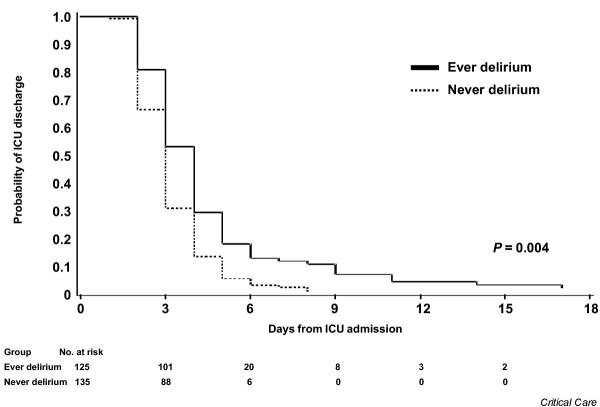
Delirium versus ICU length of stay. This Kaplan-Meier plot shows the relationship between delirium and length of stay in the ICU by classification of Ever Delirium versus Never Delirium (p = 0.004, univariate analysis).

**Figure 2:**
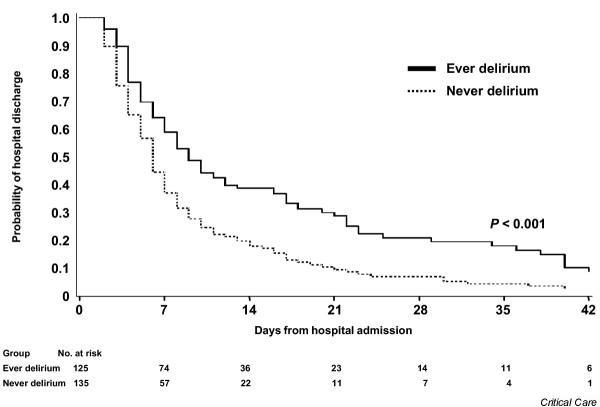
Delirium versus hospital length of stay. This Kaplan-Meier plot shows the relationship between delirium and hospital length of stay by classification of Ever Delirium versus Never Delirium (p < 0.001, univariate analysis).

Results indicate that the Ever Delirium group stayed in the ICU one day longer (median days 4; interquartile range (IQR) 3 to 5 versus 3; IQR 2 to 4) and in the hospital two days longer (median days 5; IQR 2 to 8 versus 3; IQR 2 to 6) than the Never Delirium group. A Kaplan-Meier plot for the probability of remaining in the ICU according to the clinical distinction of Ever Delirium vs Never Delirium is shown in Fig. 1. A Kaplan-Meier plot for the probability of remaining in the hospital for the same groups is shown in Fig. 2. As shown in Table 2, at any given time during their ICU stay, patients who experienced at least one episode of delirium had a 29% greater risk of remaining in the ICU even after adjusting for age, gender, race, Charlson co-morbidity score, APACHE II score, and coma (hazard ratio (HR) 1.29; 95% confidence interval (CI) 0.98–1.69, p = 0.07). Similarly, patients who experienced delirium had a 41% greater risk of remaining in the hospital after adjusting for the same covariates (HR 1.41; 95% CI 1.05–1.89, p = 0.023).

#### In-hospital mortality

**Figure 3:**
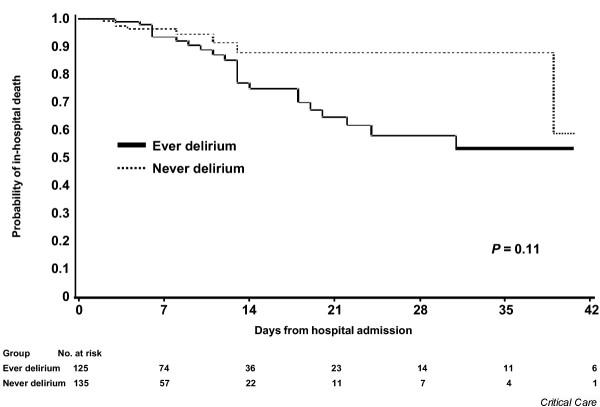
Delirium versus in-hospital mortality. This Kaplan-Meier plot shows the relationship between delirium and in-hospital mortality by classification of Ever Delirium versus Never Delirium (p = 0.11, univariate analysis).

Of the patients in the Ever Delirium group, 19% died versus 6% of the Never Delirium patients. A Kaplan-Meier plot for the probability of death according to the clinical distinction of Ever Delirium versus Never Delirium is shown in Fig. 3. Cox proportional hazards regression results indicated that delirium was not significantly associated with time to in-hospital mortality after controlling for coma status, age, gender, race, APACHE II score, and Charlson co-morbidity index (p = 0.58; Table 2).

## Discussion

Delirium developed in approximately half of the patients in our cohort, and was associated with a one day longer stay in the ICU and a two day longer stay in the hospital. This is the first investigation to document the high prevalence of delirium among a strictly non-ventilated adult ICU cohort, and to reveal its associated negative clinical outcomes. Considering the rising overall resource use and economic burden of caring for critically ill patients [25-27], our finding that ICU delirium is an independent predictor of longer stay in the hospital is of particular relevance. These data lend support to the SCCM clinical practice guideline recommendation [15] for routine monitoring of delirium for all adult ICU patients using validated tools such as the CAM-ICU, which has been validated in ventilated and non-ventilated critically ill patients [2,19].

We did not find a significant independent relationship between delirium and mortality after adjusting for multiple covariates. This may simply be a type II error due to the limited number of events, and our study was not prospectively powered to determine a definitive relationship between delirium and mortality. Furthermore, because we only followed patients until hospital death or discharge, our mortality analysis was not as comprehensive as previous reports that followed patients for 6 to 12 months [10,28]. While these ICU patients had a lower severity of illness than those in prior ICU studies isolated to ventilated patients, the myriad of data in other non-ICU populations showing delirium to be associated with prolonged stay, greater dependency of care, subsequent institutionalization, and increased mortality [3,5-7,12,28-35] would cause one to pause before assuming that our study disproves such a consistently strong association.

The dangerous and costly considerations of prolonged ICU and hospital stays shown in this cohort warrant strong consideration by multidisciplinary ICU teams. Standardized clinical monitoring of brain function (both arousal level and delirium) is in keeping with the 'systems approach' to patient assessment. Because the development of delirium is associated with untoward outcomes, one author has questioned whether or not missing the diagnosis is a medical error [36]. Considering that symptoms of ICU delirium are largely hypo- rather than hyper-active [37,38], anything short of objectively looking for delirium will result in undetected brain dysfunction. Thus, the alternative to daily monitoring for delirium is to persist with the status quo in which an estimated 60% to 80% of delirium is missed in the absence of standardized monitoring [37-41].

The strengths of this report include the unique patient population (non-ventilated ICU patients), the large number of patients enrolled (n = 261), and the consecutive enrollment process that spanned nearly a year. All data were derived from sedation scoring and delirium assessments by the bedside nurses as part of a multidisciplinary approach to care within the ICU using well-validated tools (RASS and CAM-ICU) on a frequent basis (i.e., at least once every 12 hours). Previous studies regarding the incidence of delirium have used either q-24 hour or q-weekly assessments. Study personnel performed spot checks prospectively, accuracy was confirmed [16], and data were analyzed using robust statistical methods. In fact, rather than simple logistic regression, we chose the more sophisticated approach using time-to-event analysis with Cox regression and treated both delirium and death as time-dependent covariates.

Several limitations of this study warrant comment. First of all, we did not have a tool to stratify by the severity of delirium. If such a tool had been available and employed, we may have been better able to recognize patients who were at the highest risk for negative outcomes. Currently, no validated measure to stratify the severity of delirium exists, though work in this area is ongoing. Second, a recurrent limitation in all cohort studies is that there may be unknown covariates that influence outcomes. Third, this observational investigation was not designed to prove a cause-and-effect relationship between delirium and clinical outcomes. It is certainly true that the delirium group was older and had a higher severity of illness, though our multivariable analysis was specifically designed to take these covariates into account. Ultimately, further research incorporating a randomized, prospective clinical trial focused either upon the prevention or treatment of delirium will be necessary to confirm such a relationship. Data from other investigations, however, suggest that such a cause-and-effect between delirium and negative clinical outcomes exists. For example, in response to systemic infections and injury, brain dysfunction may ensue, which will then lead to the generation of a central nervous system inflammatory response of its own. This process involves the production of specific cytokines, cell infiltration, and tissue damage [42,43]. Additionally, activation of the central nervous system's immune response is accompanied by the peripheral production of tumor necrosis factor α, interleukin 1, and interferon δ [42,44-46] that can contribute to multiple organ dysfunction syndrome. It is plausible, therefore, that the delirium experienced among our patients is not only a marker of end-organ damage, but also acts directly as a promoter of other organ system dysfunction.

## Conclusion

Nearly one out of every two non-ventilated adult ICU patients in our cohort experienced delirium. Even after adjustment for multiple covariates, delirium was associated with a longer ICU stay and was an independent predictor of a longer hospital stay. We believe that these data are clinically significant, reinforce the SCCM clinical practice guidelines for the delivery of sedation and analgesia calling for routine delirium monitoring of all patients (including those not on mechanical ventilation), and should stimulate future research in the field of delirium prevention and treatment.

## Key messages

• 	Delirium is a form of brain dysfunction known to be associated with higher mortality, cost, and long-term cognitive impairment in mechanically ventilated adults.

• 	The SCCM guidelines for sedation and analgesia recommend that ICU teams routinely monitor all ICU patients (ventilated ornot) for delirium, though little data exist for the non-ventilated group.

• 	In this prospective cohort study, delirium was detected using the CAM-ICU, which has been validated for use in both ventilated and non-ventilated patients. We found that delirium occurred in one out of every two non-ventilated ICU patients.

• 	Even after adjustment for relevant covariates, delirium was found to be an independent predictor of longer hospital stay. While univariate analysis found an association with higher mortality, that association did not reach statistical significance in the multivariable analysis.

• 	This study lends clinical relevance to adoption of delirium monitoring in all ICU patients, both those on and off mechanical ventilation.

## Abbreviations

APACHE II = Acute Physiology and Chronic Health Evaluation II; CAM-ICU = confusion assessment method for the ICU; CI = confidence interval; HR = hazard ratio; ICU = intensive care unit; IQR = interquartile range; RASS = Richmond Agitation-Sedation scale; SCCM = Society of Critical Care Medicine.

## Competing interests

The author(s) declare that they have no competing interests.

## Authors' contributions

Each author of this manuscript has: made substantial contributions to conception and design, acquisition of data, and the analysis or interpretation of data; been involved in drafting the article or revising it critically for important intellectual content; and given final approval of the submitted version to be published.

## Supplementary Material

Additional File 1 A pdf file with the Richmond Agitation-Sedation Scale. Click here for file

Additional File 2 A pdf file with the CAM-ICU Features and Descriptions Click here for file

## Figures and Tables

**Table 1:** Patient demographicsa^a^

	Ever Delirium (n = 125)	Never Delirium (n = 135)	p-value
Characteristic			
Mean age (± 1 SD; years)	56 (± 18)	49 (± 17)	0.002
Male	62 (50%)	67 (50%)	1.0
No. of Caucasians	99 (79%)	115 (85%)	0.25
APACHE II score, median (IQR)	15 (10–21)	11 (6–16)	<0.001
Charlson co-morbidity index, median (IQR)	4 (2–7)	3 (1–6)	0.079
Diagnostic category for ICU admission (%)b			
Pulmonary	29	40	
Gastrointestinal	20	21	
Metabolic	22	18	
Cardiac	7	9	
Hematology/oncology	5	4	
Neurologic	5	3	
Renal	9	2	
Other	3	3	

^a^One patient of the 261 enrolled had persistent coma and was never able to be evaluated for delirium. This patient was not included in the tables or figures. ^b^The diagnostic categories for ICU admission were recorded by the patients' medical teams as the diagnostic category most representative of the reason for ICU admission. There was no statistically significant difference between the groups in terms of admission categories (p = 0.23). Acute Physiology and Chronic Health Evaluation II (APACHE II) is a severity of illness scoring system, and these data were calculated using the most abnormal parameters during the first 24 hours following admission to the intensive care unit. APACHE II scores range from 0 (best) to 71 (worst). The Charlson co-morbidity index represents the sum of a weighted index that takes into account the number and seriousness of pre-existing comorbidities. ICU, intensive care unit; SD, standard deviation.

**Table 2:** Clinical outcomes and multivariable analysis results

	Ever Delirium (n = 125)	Never Delirium (n = 135)	Hazard ratio^a ^(95% CI)	p-value^a^
LOS in ICU^b^	4 (3,5)	3 (2,4)	1.29 (0.98–1.69)	0.07
LOS in hospital^b^	5 (2,8)	3 (2,6)	1.41 (1.05–1.89)	0.023
In-hospital mortality^c^	24 (19%)	8 (6%)	1.27 (0.54–2.98)	0.58

^a^Hazard ratios and p-values taken from multivariable Cox proportional hazards regression models adjusting for coma status, age, gender, race, APACHE II score, and Charlson co-morbidity index. ^b^Intensive care unit (ICU) and hospital lengths of stay expressed as median days with interquartile ranges. ^c^Mortality expressed as n (%). CI, confidence interval; LOS, length of stay.
