# Supplementary material for: Intensive care unit delirium is an independent predictor of longer hospital stay: a prospective analysis of 261 non-ventilated patients
Source: Crit Care. 2005 Jun 1;9(4):R375–81. doi: 10.1186/cc3729 (PMC1269454; doi:10.1186/cc3729)
Supplement: Additional File 1 — A pdf file with the Richmond Agitation-Sedation Scale. [file cc3729-S1.pdf]

ADDITIONAL FILE 1

The Richmond Agitation-Sedation Scale

Score	Term	Description	
+4	Combative	Overtly combative, violent, immediate danger to staff	
+3	Very agitated	Pulls or removes tube(s) or catheter(s); aggressive	
+2	Agitated	Frequent non-purposeful movement, fights ventilator	
+1	Restless	Anxious but movements not aggressive vigorous	
0	Alert and calm		
-1	Drowsy	Not fully alert, but has sustained awakening (eye-opening/eye contact) to *voice* (≥10 seconds)	Verbal Stimulation
-2	Light sedation	Briefly awakens with eye contact to *voice* (<10 seconds)	
-3	Moderate sedation	Movement or eye opening to *voice* (but no eye contact)	
-4	Deep sedation	No response to voice, but movement or eye opening to *physical* stimulation	Physical Stimulation
-5	Unarousable	No response to *voice or physical* stimulation	
