# Supplementary material for: Intensive care unit delirium is an independent predictor of longer hospital stay: a prospective analysis of 261 non-ventilated patients
Source: Crit Care. 2005 Jun 1;9(4):R375–81. doi: 10.1186/cc3729 (PMC1269454; doi:10.1186/cc3729)
Supplement: Additional File 2 — A pdf file with the CAM-ICU Features and Descriptions [file cc3729-S2.pdf]

ADDITIONAL FILE 2

CAM-ICU Features and Descriptions		
**1. Acute Onset or Fluctuating Course**	**Absent**	**Present**
**A**. Is there evidence of an acute change in mental status from the baseline?<br>**OR**<br>**B.** Did the (abnormal) behavior fluctuate during the past 24 hours, that is, tend to come and go, or increase and decrease in severity as evidenced by fluctuation on a sedation scale (e.g. RASS), GCS, or previous delirium assessment?		
**2. Inattention**	**Absent**	**Present**
Did the patient have difficulty focusing attention as evidenced by **scores *less than 8*** on either the auditory or visual component of the **Attention Screening Examination (ASE**)? (Instructions on next page).		
**3. Disorganized Thinking**	**Absent**	**Present**
Is there evidence of disorganized or incoherent thinking as evidenced by **incorrect answers to 2 or more of the 4 questions and/or inability to follow the commands**?<br><br>**Questions** (Alternate Set A and Set B)**:**<br>**Set A**<br>1. Will a stone float on water?<br>2. Are there fish in the sea?<br>3. Does one pound weigh more than two pounds?<br>4. Can you use a hammer to pound a nail?<br>**Set B**<br>1. Will a leaf float on water?<br>2. Are there elephants in the sea?<br>3. Do two pounds weigh more than one pound?<br>4. Can you use a hammer to cut wood?<br><br>**Other:**<br>1. Are you having any unclear thinking?<br>2. Hold up this many fingers. (Examiner holds two fingers in front of patient)<br>3. Now do the same thing with the other hand. (Not repeating the number of fingers)		
**4. Altered Level of Consciousness**	**Absent**	**Present**
Is the patient's level of consciousness anything *other than alert* such as vigilant, lethargic, or stupor? (e.g., RASS other than "0" at time of assessment)<br><br>**Alert** spontaneously fully aware of environment and interacts appropriately<br><br>**Vigilant** hyperalert<br><br>**Lethargic** drowsy but easily aroused, unaware of some elements in the environment, or not spontaneously interacting appropriately with the interviewer; becomes fully aware and appropriately interactive when prodded minimally<br><br>**Stupor** becomes incompletely aware when prodded strongly; can be aroused only by vigorous and repeated stimuli, and as soon as the stimulus ceases, stuporous subject lapse back into the unresponsive state		
**Overall CAM-ICU** (Features 1 and 2 and either Feature 3 or 4):	**Yes**	**No**
